# Substrate Uptake by TonB‐Dependent Outer Membrane Transporters

**DOI:** 10.1111/mmi.15332

**Published:** 2024-12-03

**Authors:** Volkmar Braun

**Affiliations:** ^1^ Max‐Planck‐Institute of Biology Tübingen Germany

**Keywords:** energy delivery, TonB protein, transport

## Abstract

TonB is an essential component of an energy‐generating system that powers active transport across the outer membrane (OM) of compounds that are too large or too scarce to diffuse through porins. The TonB‐dependent OM transport proteins (TBDTs) consist of β barrels forming pores that are closed by plugs. The binding of TonB to TBDTs elicits plug movement, which opens the pores and enables nutrient translocation from the cell surface into the periplasm. TonB is also involved in the uptake of certain proteins, particularly toxins, through OM proteins that differ structurally from TBDTs. TonB binds to a sequence of five residues, designated as the TonB box, which is conserved in all TBDTs. Energy from the proton motive force (pmf) of the cytoplasmic membrane is transmitted to TonB by two proteins, ExbB and ExbD. These proteins form an energy‐transmitting protein complex consisting of five ExbB proteins, forming a pore that encloses the ExbD dimer. This review discusses the structural changes that occur in TBDTs upon interaction with TonB, as well as the interaction of ExbB‐ExbD with TonB, which is required to transmit the energy of the pmf and thereby open TBDT pores. TonB facilitates import of a wide range of substrates.

## Graphical abstract

Recent findings have changed the view of the outer membrane (OM) as a protective skin to a structurally and functionally highly versatile biomembrane. It contains energy‐dependent transport systems powered by the proton motive force of the cytoplasmic membrane (CM). This review discusses the present state of insight into the novel mechanism for how the TonB protein plays a central role in the transmission of energy from the CM into the OM during transport.

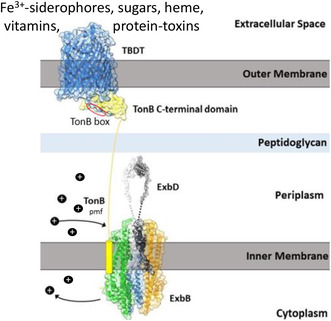

## 1 Introduction

Gram‐negative bacteria, such as *Escherichia coli*, are protected by an outer membrane (OM). Despite open porin channels in orders of 10^5^ per cell, small deleterious compounds in the environment have little access to cells (Nikaido 2003). They are excluded by size or by active export systems. In contrast, nutrients must be able to cross the OM. This is particularly evident for nutrients that are scarcely available to cells, such as Fe. Although present at rather high concentrations under anaerobic conditions, Fe^2+^ is oxidized to Fe^3+^ under aerobic conditions and forms insoluble hydroxy‐aquo complexes, making it biologically unavailable. To overcome Fe deprivation, bacteria have developed a specific Fe^3+^‐solubilization strategy in which Fe is synthesized and released into the growth medium as Fe^3+^‐complexing compounds, designated siderophores, or they use siderophores produced by fungi (Neilands 1981). The Fe^3+^‐siderophore complexes are then taken up into cells by highly specific transport systems. In contrast to most nutrient uptake systems, energy is not only required for Fe^3+^‐siderophore transport across the cytoplasmic membrane (CM) but also across the OM. In addition to Fe^3+^siderophores, a large variety of compounds, including metal ions, vitamin B_12_, glycans, and proteins, are actively transported through the OM (Table 1).

**TABLE 1:** TonB‐dependent outer membrane transport proteins (TBDTs).

Organism	Gene	PDB	Function	Reference
*E. coli*	*fhu*A	1BY3 1FCP	Fe^3+^‐hydroxamate transporter, receptor for phages T1, T5, φ80, UC‐1 colicin M	Ferguson et al. (1998), Locher et al. (1998)
*E. coli*	*fepA*	1FEP	Fe^3+^‐enterobactin transporter, receptor for colicins B and D	Buchanan et al. (1999)
*E. coli*	*fecA*	1KMO 1PNZ	Fe^3+^‐citrate transport, ECF transcription regulator	Ferguson et al. (2002), Yue, Grizot, and Buchanan (2003)
*E. coli*	*btuB*	1NQE	Vitamin B_12_ transporter. receptor for colicins	Chimento et al. (2003)
*P. aeruginosa*	*fpvA*	1XKH	Fe^3+^pyoverdine transporter	Wirth et al. (2007)
*Pseudomonas aeruginosa*	*foxA*	PDB6196	Fe^3+^ ferrioxamine B transport, ECF transcription regulator	Josts, Veith, and Tidow (2019)
*Caulobacter crescentus*	*malA*	AF‐A0A0H3CW8‐F1	Maltodextrin transport	Neugebauer et al. (2005)
*Caulobacter crescentus*	*nagA*	AF‐A0A0H3C6W8‐F1	N‐acetyl‐glucosamine transport	Eisenbeis et al. (2008)
*Bacteroides*	*susC*	AF‐Q8A1G1‐F1	Glycan transport	Glenwright et al. (2017)
*Pseudomonas*	*fpv*A‐1	1XXH	Fe^3+^ pyoverdine transport Pyocin receptor	McCaughey et al. (2016)
*E. coli*	*yncD*	6VB1	Pyrrocholin‐quinone transport	Hantke and Friz (2022)
*Myxococcus xanthus*	*popC*	Q1DFT5	Secretion of the Oar protein	Gómez‐Santos et al. (2019)
*Serratia marcescens*	*hasR*	3CSL	heme transport	Wandersman and Delepelaire (2012)

**FIGURE 1:**
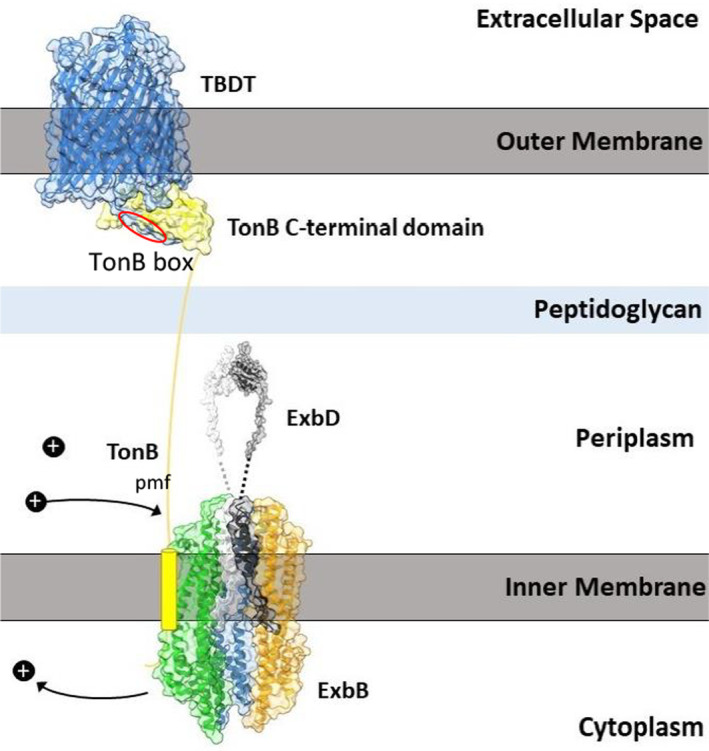
Illustration of the subcellular localization, structure and interaction of the TonB‐ExbB‐ExbD energy transfer complex from the cytoplasmic (inner)membrane across the periplasm to the TonB‐dependent outer membrane transport protein (TBDT). The proton motive force (pmf) derived from the higher concentration of protons in the periplasm than in the cytoplasm delivers the energy to open the pore in the TBDT. ExbB consists of five identical subunits, colored purple, pink, green, blue, and orange (PDB:6TY) which form a cylinder with a central pore in which an ExbD dimer (black and white cylinders) (PDB:2PFU) is located. Two of the ExbB monomers were omitted to better visualize the ExbD dimer. The periplasmic part of ExbD is disordered and therefore represented by black and white dashed lines. The transmembrane domain of TonB (yellow) is tentatively attached to ExbB since the TonB structure could not be resolved by X‐ray analysis except the C‐terminal globular domain. Energized TonB binds by its C‐terminal domain to the TonB box (marked red) of the TMDT and alters the structure of the TMDT that the substrates which are bound to the TMDT at the cell surface are released and diffuse through the opened pore of the TMDT into the periplasm. The figure does not take into account the known extensive interaction of TonB with ExbD and the binding of ExbD to the peptidoglycan (see text). Figure reproduced and changed from Braun et al. (2023) with permission.

**FIGURE 2:**
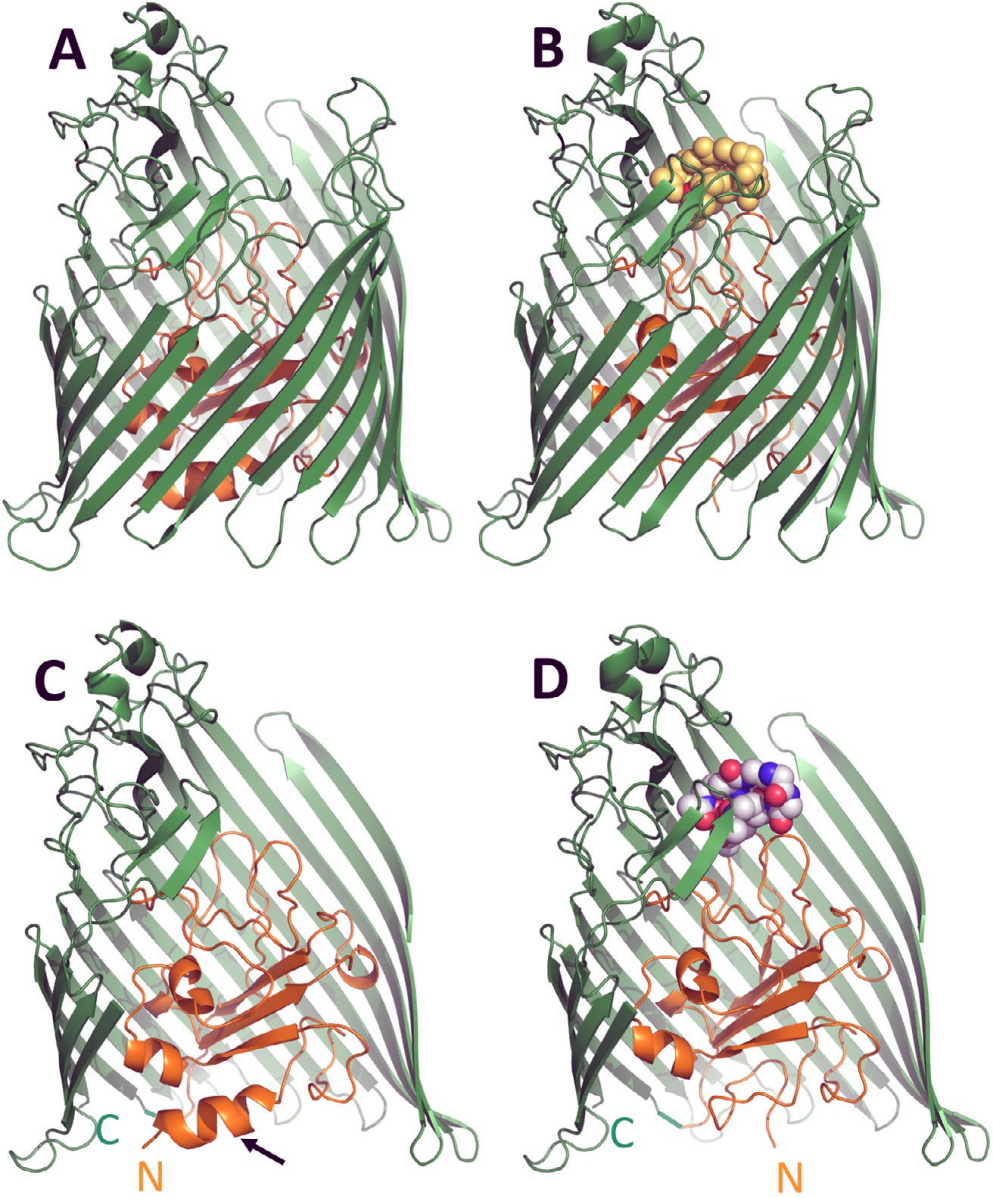
Crystal structure of the *E. coli* FhuA protein, unliganded (A) and liganded (B) with ferrichrome (yellow with the complexed iron ion in red). FhuA forms a β barrel (green) with a plug (brown) inside the pore of the barrel. In the lower panel (C, D) a segment comprising residues 478 to 657 was removed to improve visualization of the plug. The binding site of ferrichrome is located close to the cell surface at the apical interface of three turns of the plug domain and two loops of the barrel. Binding of ferrichrome induces unfolding of helix H1 (marked by an arrow) close to the periplasm and displacement of the corresponding segment to the opposite side of the barrel wall. N and C mark the N‐terminal and C‐terminal end of FhuA. The figure was obtained from Kaspar Locher (Locher et al. 1998).

It is difficult to investigate the mechanism of active energy‐consuming transport across the OM. Active transport requires energy; however, no energy source is known for the OM. The next physical energy‐generating system is the proton motive force (pmf) of the CM. However, how does the pmf energize the transport across the OM? The first insights were gained from studies on phage infection and valuable models were derived from the crystal structures of OM receptor proteins (Figures 1 and 2), mutant analysis, and various methods of spectroscopy.

The energy‐coupled functions of the OM were first proposed in phage infection studies (Garen and Puck 1951). The infection of *E. coli* by phage T1 requires actively metabolizing cells, and the initial irreversible binding of T1 to *E. coli* cells is energy‐dependent. Later, the pmf of the CM was shown to serve as an energy source, which posed the question of how energy could be transmitted from the CM to the OM (Hancock and Braun 1976). Shortly after these initial studies, colicins, protein toxins produced by some strains of *E. coli* (Cascales et al. 2007) were shown to enter prey *E. coli* strains, raising the question of how proteins with a size of up to 80 kDa can cross the OM and CM. Phages and colicins are used in genetic studies because stable resistant mutants can be conveniently isolated. Since these initial observations, substantial progress has been made in understanding energy‐dependent transport across the OM. However, fundamental understanding regarding the mechanisms of energization and translocation of substrates across the OM has not been achieved. This study intends to define the state‐of‐the‐art research by discussing the results and the models derived from the most advanced systems. A short history excursion provides an impression of how the field has developed.

## 2 Identification of the *tonB* Gene

Initially, studying bacterial genetics was a challenging task. The great Dutch bacteriologist Martinus Beijerinck wrote in 1901: “Though the culture of microbes, compared to that of higher plants and animals, is subject to many difficulties, it cannot be denied that these, once mastered, microbes are an extremely useful material for the investigation of the laws of heredity and variability” (Beijerinck 1900). The difficulties arose from the misconception that bacterial cultures, and not bacterial cells, were considered organisms (Summers 1991). Cultures were frequently derived from medical isolates and contained a mixture of strains. However, when single colonies obtained by plating on solid media were used, variants appeared after several days of growth. Hadley wrote “cocci become rods and rods cocci or spirals; forms of growth change overnight; motility is lost or regained; fermentation reactions are modified by time and opportunity; spore formers become sporeless; hemolytic activities come and go; capsulated bacteria lose their capsules, and capsules are gained by non‐capsulated forms; antigenic power vanishes and reappears; cultures becomes spontaneously agglutinative or fail of agglutination; virulent cultures become harmless and harmless cultures virulent” (Hadley 1927). Early attempts at studying bacterial genetics yielded irreproducible results. The term mutation was rarely used; instead, it referred to colony variation or dissociation. In contrast, the resistance of cells to phages was a stable reproducible marker and was used by Luria & Delbrück in their seminal paper on “Mutations of bacteria from virus sensitivity to virus resistance” in which they concluded that resistant bacteria arise by mutations of sensitive cells (Luria and Delbrück 1943). They obtained two types of resistant bacteria: one type was resistant to phages T1 and T5, and the other was resistant to T1 (they used another nomenclature; however, in this review, the current nomenclature will be used). Mutations in the *tonB* gene conferred T1 resistance, and T1 and T5 resistance was caused by mutations in the *tonA* gene. The *tonB* gene was ultimately identified when its nucleotide sequence was determined (Postle and Good 1983).

Phage T1 adsorbed to the *E. coli* cells in two steps. The first step was reversible, in that no infection occurred, whereas the second step was irreversible, after which the cells were infected and the infective phages could no longer be eluted from the cells by dilution or agitation. Irreversible adsorption can be inhibited by low temperatures, sodium azide, heat, or ultraviolet light which indicates a requirement for energy. T1 is adsorbed and desorbed reversibly in *tonB* mutants (Garen 1954). These observations suggested that a reaction occurred at the cell surface that required both TonB and energy. It was subsequently demonstrated that the energy source was the pmf across the CM (Hancock and Braun 1976). This finding raised the question of how energy is transferred from the CM to the OM and how TonB changes the activity of TonA (later named FhuA). In contrast, *tonB‐*independent infection with phage T5 did not require energy. Unlike T1, T5 adsorbs irreversibly to isolated membranes and releases its DNA (Weidel 1958), provided that it contains the TonA (FhuA) protein (Braun, Schaller, and Wolff 1973).

## 3 Discovery of the TonB Function

It took 26 years of research to uncover the physiological functions of TonB. The *tonB* mutants require high Fe concentrations in the growth medium (Wang and Newton 1971). They transport ferric iron with a *K*_*m*_ that is 10‐fold higher than that of wild‐type cells. In an attempt to isolate *tonB* mutants defective in ferrichrome‐mediated Fe uptake using albomycin, an antibiotic with a structure similar to that of the siderophore ferrichrome (Ferguson et al. 2000), not only a single t*onB* mutant but 32 *tonA* mutants were unexpectedly isolated (Hantke and Braun 1975). Siderophores are secreted by bacteria and fungi and have high affinity and specificity for Fe^3+^ (Neilands 1981). Subsequent isolates that were resistant to phages T1 and T5 were unable to take up Fe complexed with ferrichrome. In addition, ferrichrome protected cells from infection by phage φ80, which uses the same cell receptor as T1 (Luckey, Wayne, and Neilands 1975). At the time of these experiments, *tonA* was known to encode a protein of 80 kDa, which had already been purified to electrophoretic homogeneity and was localized in the OM (Braun, Schaller, and Wolff 1973). T5 was inactivated by the purified TonA protein. Colicin M (Cma), which uses the TonA receptor as an entry site for cells, prevents the inactivation of T5 by purified TonA. In contrast, a TonA protein variant isolated from a *tonA* point mutant resistant to T5 and Cma failed to inactivate T5 and Cma. These biochemical results were supported by previous genetic data that showed that *tonA* mutants were resistant to phage T1, φ80, and Cma (Graham and Stocker 1977). These results clearly defined the *tonA* gene product as a phage and colicin receptor that transports ferrichrome and albomycin across the OM.

## 4 TBDT Transport Proteins

### 4.1 Structure of the FhuA (TonA) Protein

TonA was renamed FhuA in accordance with the designation of *fhuBCDE* genes, which encode proteins that transport ferrichrome across the CM of *E. coli* (Kadner et al. 1980). FhuA was the first isolated pure TBDT (Braun, Schaller, and Wolff 1973) and the first TBDT with a known crystal structure that was determined by two independent groups (Ferguson et al. 1998; Locher et al. 1998). This will be discussed first, and the other known crystal structures (Table 1) will be compared with FhuA. FhuA is a multifunctional protein that serves as a binding site for various phages, the toxic proteins microcin J25 and Cma, and it transports the antibiotics albomycin and rifamycin CGP 4832 (Table 1). It consists of a β barrel composed of 22 antiparallel β strands (residues 160–714) forming a pore that is closed by a plug (residues 19–159), which itself is composed of a four‐stranded β sheet with interdispersed α helices and connecting loops (Figure 2). Residues 1–18 are disordered and localized in the periplasm. Ferrichrome perfectly fits into the extracellular pocket approximately 20 Å beyond the OM surface (Figure 2). Ferrichrome is bound by three plug residues (R81, Y116, and G99) and two barrel residues (Y244 and Y315), which results in strong binding (*K*_*d*_ 0.1 μM) (Locher and Rosenbusch 1997).

The comparison of the FhuA crystal structures in the free form and loaded with ferrichrome (Figure 2), revealed very little change in the barrel except for large conformational transitions in the plug. Specifically, the switch helix, an α helix containing residues 24–29 and located close to the periplasm (marked by an arrow), was unfolded in the ligand‐bound form. Tryptophan 22 displaced 17 Å to the opposite side of the barrel wall and occluded the periplasmic end of the channel‐forming region. Unwinding of the switch helix functions as a tightly regulated transmembrane signal that distinguishes between occupied and unoccupied FhuAs (Ferguson and Deisenhofer 2002).

### 4.2 TonB Binds to the TonB Box of TBDTs

**FIGURE 3:**
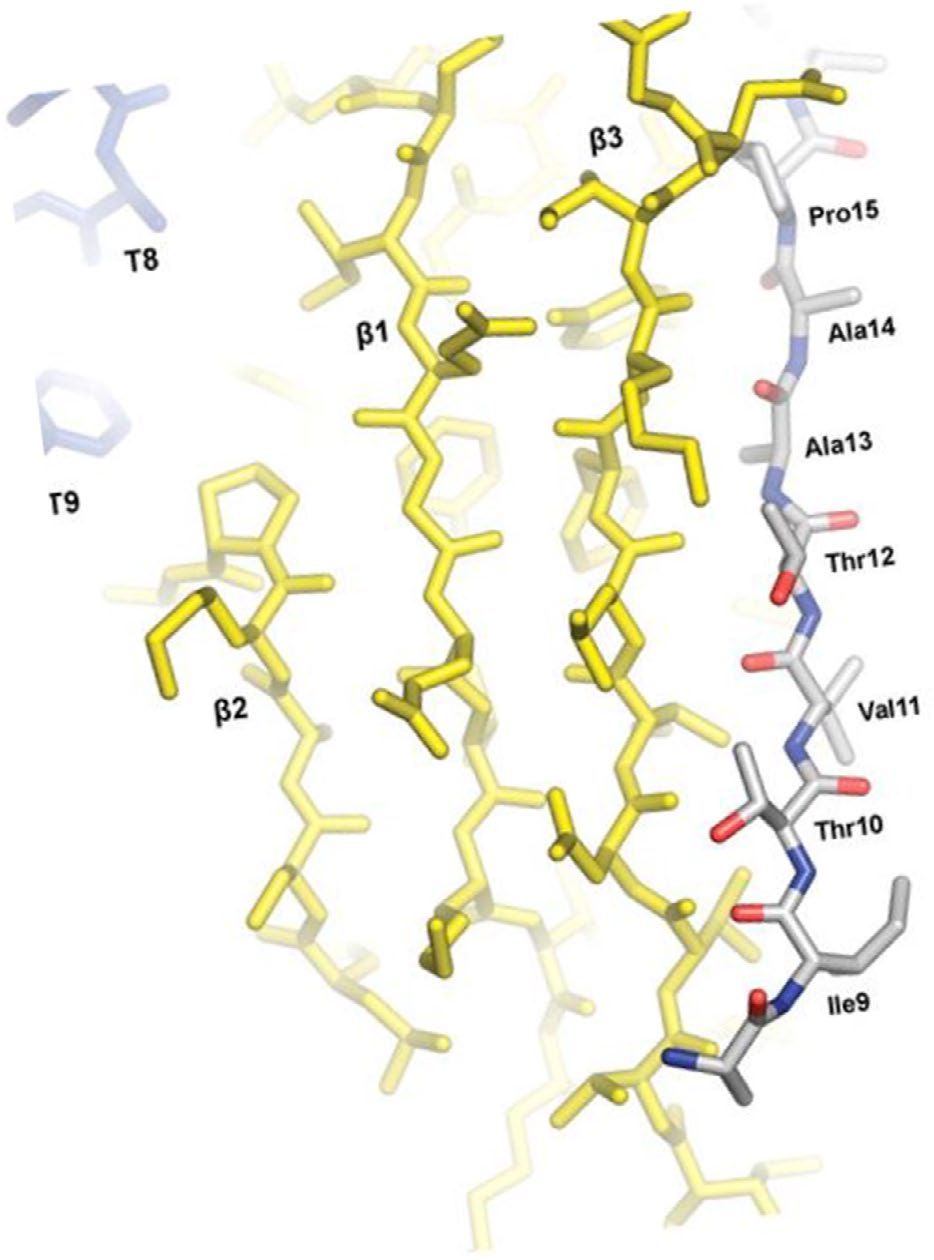
Interprotein β sheet formed between the FhuA TonB box (colored blue) and the central β sheet of the C‐terminal domain of TonB (yellow), as derived from the crystal structure of the FhuA‐TonB (residues 33 to 239) complex. FhuA and TonB residues are shown as sticks. TonB strands β1 to β3 are labeled. FhuA periplasmic turns 8 and 9 (T8, T9) are labeled for reference. The figure was reproduced with permission from Pawelek et al. (2006).

The pronounced allosteric transitions induced by the binding of ferrichrome to FhuA possibly recruit TonB to residues 7–11 of FhuA, the principal site of interaction between FhuA and TonB, designated as the TonB box present in all TBDTs (Heller, Mann, and Kadner 1985) and in all colicins that are taken up by a TonB‐dependent mechanism (Schramm et al. 1987). Therefore, the TonB box is the preferred region for studies aimed at determining the mechanism through which TonB initiates TBDT‐mediated uptake of compounds across the OM. In the crystal structure of FhuA in complex with the C‐terminal fragment of TonB (residues 33–239) (Pawelek et al. 2006) the TonB box of FhuA (residues 7–11) forms a parallel β interaction with β3 of the TonB C‐terminal domain resulting in the formation of an interprotein β sheet β augmentation (Figure 3). The TonB box occupied approximately half of the periplasm‐exposed surface area of FhuA and occluded the barrel lumen.

Biochemical studies of *fhuA* and *tonB* mutants were performed to validate the predictions derived from the FhuA structure. The *fhuA* mutants, with a mutation in the TonB box (V11D) or a complete deletion, lacked all energy‐dependent functions of FhuA but retained energy‐independent T5 infection (Endriss et al. 2003). T5 sensitivity analysis showed that the mutations did not abolish the proper insertion of active FhuA into the OM. The amino acid replacement I9P was predicted to inactivate FhuA but unexpectedly, the mutant protein retained 18% of the ferrichrome transport activity and the strain carrying FhuA I9P remained sensitive to T1 and φ80. Notably, the amino acid replacements I9P and V11D and deletion of the switch helix (residues 23–30) displayed 30% stronger ferrichrome binding than that of wild‐type FhuA. These mutations were located distantly from the ferrichrome‐binding sites, thus demonstrating long‐range structural transitions from the plug to the cell surface. For the uptake of ferrichrome, the plug must be displaced and the geometry of the residues at the initial binding site must be changed to release ferrichrome into the pore. In contrast, the I9P mutation of BtuB another TBDT (Table 1) completely abolished vitamin B_12_ uptake, which is usually observed with other TBDTs mutants. The activity of the TonB box depends less on the individual amino acid side chains than on the local secondary structure (Gudmundsdottir et al. 1989).

To examine the structural changes in the TonB box of FhuA in response to the binding of ferrichrome and TonB, electron paramagnetic resonance spectroscopy (EPR) was performed, which revealed dynamic protein–protein coupling between FhuA and TonB (Sarver et al. 2018). The TonB box of unliganded purified FhuA reconstituted into liposomes assumed a broad range of positions and multiple conformational states, which were altered by loading with ferrichrome; however, the TonB box did not extend further into the periplasm. After addition of a soluble TonB fragment (residues 103–239) the TonB‐FhuA complex remained heterogeneous and flexible, indicating a lack of strong contacts between TonB and the plug and barrel. The affinity of TonB for FhuA increases in the presence of ferrichrome (Freed et al. 2013; Moeck, Coulton, and Postle 1997; Moeck and Letellier 2001; Khursigara et al. 2004), however, the EPR spectra showed no substrate‐induced changes in the local dynamics of the TonB box. Binding of the TonB (103–239) fragment did not change the flexibility of the TonB box in the presence or absence of ferrichrome. These findings contrast with the crystal structure of FhuA‐TonB (residues 155–239) in which TonB stabilizes the amino‐terminal residues of FhuA, including the TonB box (Pawelek et al. 2006). They also differ from the EPR data for BtuB (see below), in which substrate binding shifts the conformational equilibrium in the TonB box to favor an unfolded state that projects the TonB box into the periplasmic space.

Residues R93 and R133 are conserved among TBDTs and are predicted to fix the plug to the β barrel by forming a salt bridge to the conserved residues E522 and E571 of the β barrel. Disruption of the salt bridge by replacing glutamate residues with arginine inactivated all FhuA activities. Inactivation did not result from repulsion between the two arginine residues, R93 and R522, because the replacement of R93 with leucine, R93L E522R, also rendered FhuA inactive (Endriss et al. 2003). Inhibition of plug movement by the introduction of cysteines and fixation of position 27 downstream of the TonB box to position 533 of the barrel abolished ferrichrome transport, which was restored by the reduction of the disulfide bond. These data indicated that the plug must be flexible for TonB‐coupled FhuA activity. Deletion of residues 24–31 which included the switch helix, reduced ferrichrome transport activity to 79% of FhuA wild type and retained full sensitivity to Cma and phages T1, φ80, and T5.

The experimentally determined affinity values of FhuA for TonB depend on the conditions used. For example, in a detergent solution, FhuA binds to TonB with high affinity, approximately *K*_*D*_ 20 nM, irrespective of the presence of ferricrocin (Khursigara et al. 2004). Repetition of these experiments with FhuA, reconstituted in nanodisks, revealed a tenfold lower affinity, approximately *K*_*D*_ 200 nM (Mills et al. 2014). Isothermal titration calorimetry of FhuA in the nanodisks with TonB in the presence of ferricrocin revealed an equilibrium affinity of approximately 200 nM and a binding stoichiometry of 0.98. No heat was generated in the absence of ferricrocin.

### 4.3 BtuB Transport Protein

The first OMP that was shown to transport a substrate across the OM in an energy‐dependent fashion was the vitamin B_12_ transporter BtuB (Di Girolamo and Bradbeer 1971). Transport occurs in two phases: an initial rapid energy‐independent phase and a secondary slower energy‐dependent phase. The initial process showed saturation kinetics with a *V*_max_ 56 molecules per second per cell and a *K*_*m*_ of 5 nm. It is now known that the initial phase corresponds to binding of vitamin B_12_ to BtuB and the second phase corresponds to transport across the OM and CM into the cytoplasm. The crystal structures revealed an ordered TonB box that underwent a subtle conformational change in the presence of vitamin B_12_ and remained folded within the plug (Chimento et al. 2003). In contrast, EPR spectroscopy of purified spin‐labeled BtuB revealed that binding of vitamin B_12_ to BtuB shifts a conformational equilibrium in the TonB box in favor of an unfolded state (Fanucci et al. 2003) that projects the TonB box approximately 20–30 Å into the periplasmic space (Xu et al. 2006) which may provide a signal that initiates the interactions between BtuB and TonB (Cadieux et al. 2003). The EPR of spin‐labeled cysteine residues revealed that upon vitamin B_12_ binding, the TonB box transitions from a folded structure fixed to the barrel to an extended, disordered, dynamic structure (Merianos et al. 2000). Site‐directed disulfide bonding within intact cells showed that the TonB box approached residues at position 160 of TonB in a highly oriented and specific manner to form BtuB‐TonB heterodimers.

BtuB also serves as a receptor through which colicin E3 enters cells (Cascales et al. 2007). E3 belongs to the A‐class of colicins, which are taken up by cells via the Tol system. E3 is composed of three structurally separated domains: an N‐terminal translocation domain, a central receptor‐binding domain, and a C‐terminal activity domain that specifically cleaves 16SRNA. EPR spectroscopy of the purified BtuB protein showed that the E3 receptor‐binding fragment reversed vitamin B_12−_induced undocking of the TonB box (Fanucci et al. 2003).

Vitamin B_12_ transport studies have revealed that the results obtained using isolated proteins can differ from those obtained using living cells. For example, the solutes used in protein crystallization inhibit strong order‐to‐disorder transitions in the TonB box upon vitamin B_12_ binding (Kim, Fanucci, and Cafiso 2007). The effect of the methods used to prepare BtuB on the results was considered in the pulse EPR measurements of intact cells (Nilaweera et al. 2021). Substrate binding altered the C‐terminal region of the plug and shifted loop 3 by 2 nm toward the periplasm. Structural transitions are regulated by the ionic bond between Arg14 of the plug and Asp316 of the barrel, which breaks upon binding of vitamin B_12_ and TonB. These structural changes did not occur when purified BtuB was placed in an artificial membrane.

### 4.4 FepA Transport Protein

FepA transports Fe‐loaded enterobactin (FeEnt) across the OM of *E. coli* in a TonB‐dependent manner (Table 1). The crystal structure of FepA was similar to that of FhuA (Buchanan et al. 1999). A globular N‐terminal domain (residues 1–153) folds into the pore of the β barrel (residues 154–724) and prevents the flow of small molecules through the pore in both directions. FeEnt binds within 1 or 2 s to loops. Two loops extend from the center of the pore toward the extracellular loops of the barrel and participate in Fe‐Ent binding and signaling across the plug into the periplasm. Fe‐Ent binding closes the FepA pore on the extracellular side and opens a channel on the periplasmic side by binding the FepA TonB box (residues 12–14) to TonB.

The isolated plug behaved as an unfolded protein and bound ferric enterobactin with an affinity of 5 μM, 100‐fold lower than that of intact FepA (Usher et al. 2001). This finding does not support the hypothesis that the plug is pulled out of the barrel intact, but rather favors a model in which the plug rearranges within the barrel to form a channel. When cysteine residues were genetically introduced into the plug and the C‐terminal transmembrane β barrel (Majumdar et al. 2020), intraplug disulfide bridges prevented ferric enterobactin transport whereas most disulfide bonds between the plug and the barrel did not affect transport. These findings indicate that conformational rearrangements within the plug are required for transport and suggest that the plug may remain in the pore and not disengage from it.

Spectroscopic analyses of fluorophore‐labeled FepA revealed the dynamic actions of surface loops during the binding and transport of ferric enterobactin (Smallwood et al. 2014). Introduction of cysteine residues in the surface loops enabled labeling of FepA with fluorescein‐5‐maleimide (FM) and revealed that the protein was evenly distributed across the entire cell surface. The transport of FeEnt resulted in fluorescence quenching and subsequent recovery as a result of Fe‐Ent uptake from the media. In contrast to the appearance of FM‐labeled FepA and FeEnt transport across the entire cell surface, including the pole regions, green fluorescence protein (GFP)‐tagged TonB was not observed in the pole region; however, fluorescence polarization measurements demonstrated the motion of GFP‐TonB, which required pmf and ExbB‐ ExbD (Jordan et al. 2013).

The discrepancy between the number of TonB molecules per cell (approximately 300 under Fe‐sufficient growth conditions, 1000 under Fe‐limiting conditions) and the number of FepA molecules (500 under Fe sufficient conditions and 14,000 under Fe limiting conditions) (Higgs, Larsen, and Postle 2002) raises the question of how many FepA molecules transport Fe‐Ent and how the transport‐proficient FepA molecules are selected by TonB. Does the low rate of FeEnt uptake, approximately kcat 5 min^−1^ (Kaserer et al. 2008) result from the fact that only a fraction of FepA transports FeEnt? This is a fundamental problem for all TonB‐TBDT interactions. To answer this question, a post‐uptake binding assay was devised (Newton et al. 2010) that allowed the quantity and fraction of active FepA proteins to be determined. If the FepA receptors are saturated with ^56^FeEnt at 0°C and then shifted to 37°C, the FepA proteins that transport FeEnt become vacant and available for the new binding of ^59^FeEnt. This experiment revealed that all FepA proteins with bound ^56^FeEnt transported ^56^FeEnt and adsorbed it from the medium. The maximum rate, *V*_max_, of FeEnt uptake into the periplasm by the chromosomally encoded transport system was 78 pmol/min/10^9^ cells in the first 10 min (Newton et al. 2010).

The cell‐associated Fe concentration is approximately 10^6^ ions per cell (Newton et al. 2010). The number of FepA molecules per cell varies strongly, depending on the Fe concentration in the growth medium (Higgs, Larsen, and Postle 2002; Kaserer et al. 2008) Taking 10^4^ as the average value for logarithmically growing cells, a single FepA molecule would transport only 100 FeEnt molecules in a 60 min generation time. However, because there may only be 100 TonB molecules available for FeEnt transport, the FeEnt flux through FepA would be of the order of 10^4^ in 60 min. This consideration is based on the assumption that after each transfer of a FeEnt molecule, TonB dissociates from FepA and associates again for the next transfer. However, if TonB remains associated with FepA for many transport cycles, the transport capacity of a single FepA molecule could be much higher. The findings presented above (Smallwood et al. 2014; Newton et al. 2010) that all FepA molecules on the cell surface transport FeEnt are not compatible with the low number of TonB molecules unless TonB is rapidly exchanged between FepA molecules.

### 4.5 TBDT Transport and Regulatory Proteins

#### 4.5.1 FecA Protein

**FIGURE 4:**
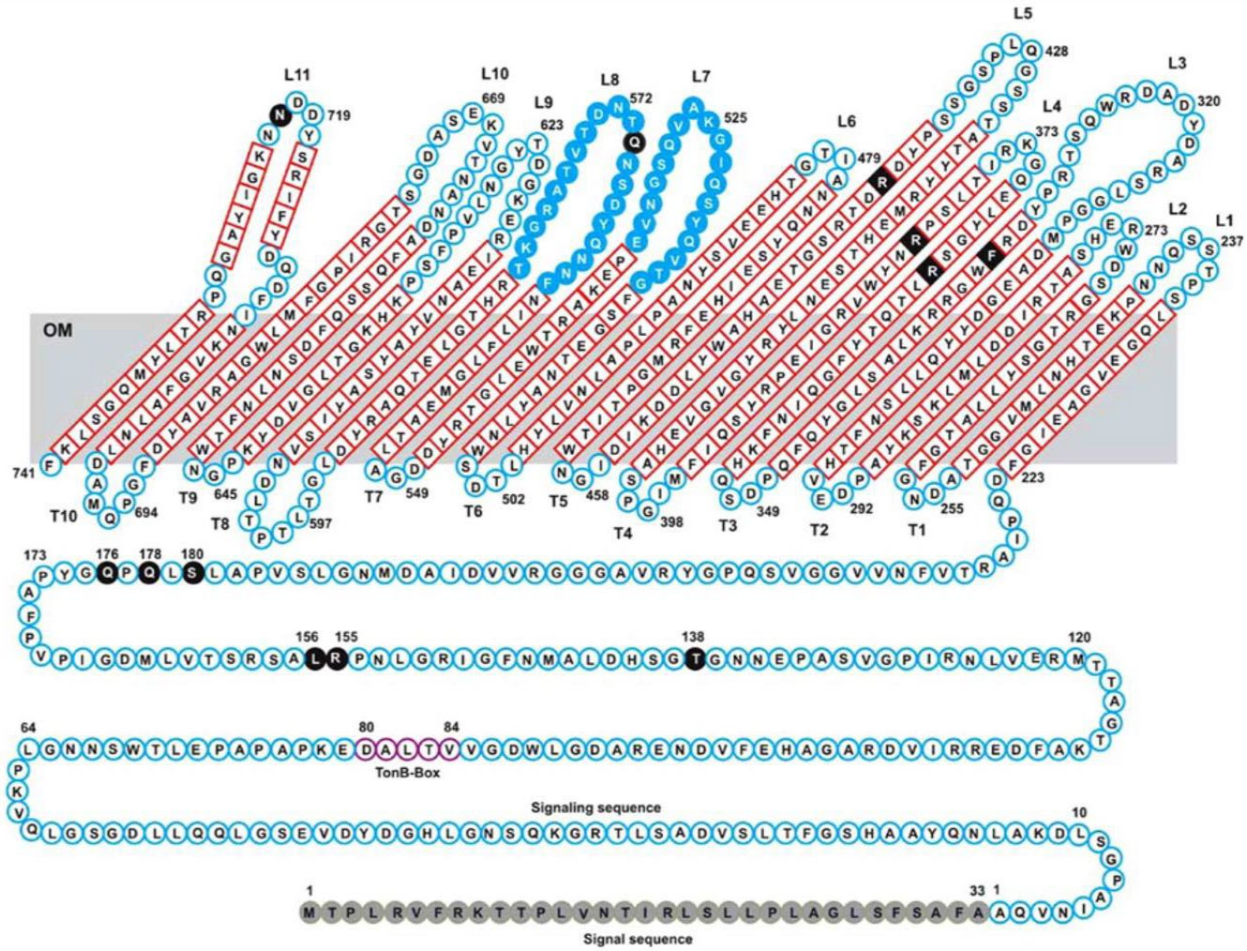
Topology of the FecA polypeptide chain across the outer membrane of *E. coli*. The transmembrane antiparallel β‐strands of the unfolded β barrel are shown in red; loops 7 and 8, which strongly move upon binding of dinuclear ferric citrate are shaded blue and the proposed ferric citrate binding residues are shaded black. The signal peptide (residues 1–33) released upon secretion of FecA across the cytoplasmic membrane, the signaling sequence (residues 1–79 of the mature protein) involved in transcription regulation by interaction with FecR, and the TonB box are indicated. The globular plug domain (residues 80–221) inserted in the β barrel is shown as linear sequence. Reproduced from Braun and Mahren (2005).

FecA differs from most of the TBDTs (Koebnik 2005) in that it not only transports ferric citrate into the periplasm but also initiates transcription of the *fec* transport genes by means of an additional domain (residues 1–79), which is a designated signaling domain and located in front of the TonB box (residues 80–84; Figure 4) (Braun, Hartmann, and Hantke 2022). In the periplasm, the signaling domain interacts with the FecR protein, which extends into the cytoplasm, where its N‐terminal fragment activates the FecI sigma factor, resulting in the transcription of *fecABCDE* transport genes (Braun, Hartmann, and Hantke 2022). Signaling by ferric citrate‐bound FecA requires the same energization as that required for ferric citrate transport across the OM via TonB, ExbB, and ExbD. Crystallographic analysis of FecA did not resolve the structure of the signaling domain (Ferguson et al. 2002; Yue, Grizot, and Buchanan 2003), but nuclear magnetic resonance (NMR) analysis revealed the structure in solution of the signaling domain with an additional 17 residues extending into the plug domain (García‐Herrero and Vogel 2005). It consists of two different regions: a well‐defined folded domain (residues 1–74) and an extended flexible tail (residues 75–96). The extra N‐terminus places the predicted TonB box of FecA in unusual positions at residues 80–84 (Figure 4). Deletion of the TonB box inactivates FecA transport and signaling (Ogierman and Braun 2003). Physical interaction between the TonB box of FecA and the region at and around residue 160 of TonB has been demonstrated by in vivo site‐specific disulfide bond formation between the cysteine derivatives of FecA and TonB (Ogierman and Braun 2003). Unexpectedly, ferric citrate did not increase the formation of disulfide crosslinks, and the in vivo site‐specific interactions between TonB and FecA did not require TonB to be in an energized state.

EPR spectroscopy of spin‐labeled FecA reconstituted into 1,2‐dilauroyl‐sn‐glycero‐3‐phosphocholine (*DLPC*) lipid bilayers revealed that the signaling domain changed its conformation, but its local folds and dynamics were not altered by ferric citrate or TonB (residues 33–239) (Mokdad et al. 2012). In the absence of ferric citrate, the signaling domain was positioned at the periplasmic surface of FecA, where it interacted with the TonB box and blocked its access to the periplasm, thereby sterically inhibiting the interaction of TonB with the TonB box. Binding of ferric citrate rotates the signaling domain and exposes the TonB box, resulting in a disordered transition of the TonB box that facilitates interaction with TonB. The TonB fragment 33–239 bound to FecA displaced the signaling domain to the barrel without further movement into the periplasm. Interaction of FecA with FecR requires exposure of the signaling domain in the periplasm to enable contact with FecR and transcription initiation of the *fec* transport genes.

The deletion of the signaling domain results in regulatory‐deficient FecA mutants that are fully transport‐competent (Härle et al. 1995). Point mutants in the signaling domain displayed approximately 10% of the wild‐type transcriptional capacity, which was not regulated by FeCit. One exceptional mutant initiated transcription independently of ferric citrate and TonB but required TonB for ferric citrate transport. The properties of this mutant suggest that TonB‐dependent signaling mechanistically differs from TonB‐dependent transport.

#### 4.5.2 FpvA Crystal Structure With Signaling Domain

**FIGURE 5:**
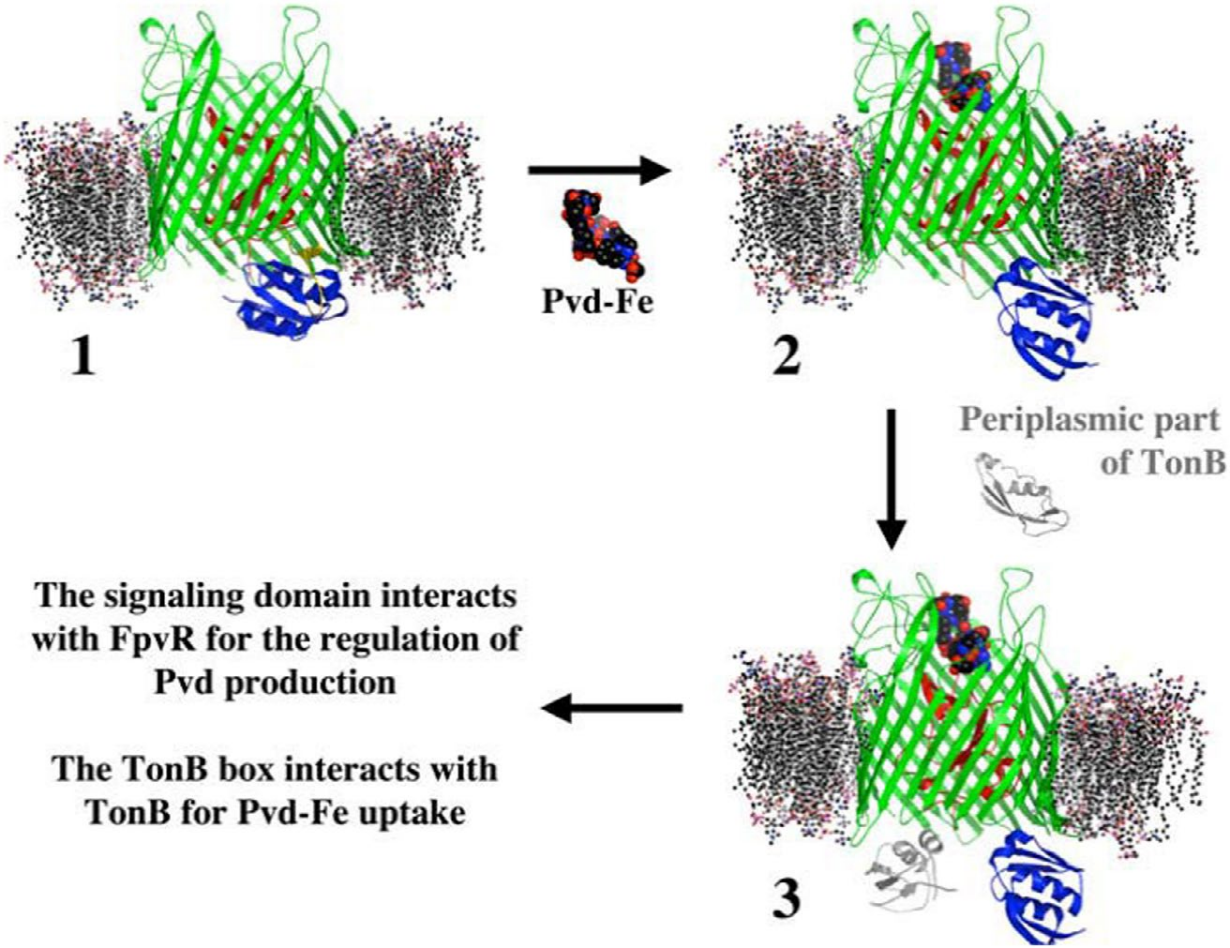
Crystal structure of the FpvA outer membrane transport protein of *Pseudomonas aeruginosa*. FpvA is composed of a β barrel (green) with a plug (red) inside. The signaling domain involved in transcription initiation of the *fpvA* gene and pyoverdine synthesis genes is colored blue. The chromophore of pyoverdine is shown in black‐blue‐red blue, its peptide moiety in yellow and iron in magenta. The figure is reproduced from Schalk, Lamont, and Cobessi (2009) with permission.

FpvA of *Pseudomonas aeruginosa* transports ferric pyoverdine (Fe‐Pyo) and regulates pyoverdine synthesis and transport (Wirth et al. 2007; Schalk and Perraud 2023) by a mechanism similar to FecA regulation of the *fec* operon (Table 1). Determination of the crystal structure of unloaded FpvA revealed complete TBDT, including the signaling domain (Figure 5) (Brillet et al. 2007). The mixed three‐stranded *β* sheet of the signaling domain formed a four‐stranded β sheet with the β strand containing the TonB box residues. The signaling domain is absent in the crystal structure of FpvA loaded with Fe‐free pyoverdine (Wirth et al. 2007) (Figure 5). Binding of Fe‐Pyo to FpvA conferred flexibility to the N‐terminal region, such that the TonB box did not show electron density. The Fe‐Pyo‐induced transitions in FpvA reduced the affinity of the signaling domain for the TonB box, with the consequence that TonB binding to the TonB box was favored.

#### 4.5.3 FoxA Crystal Structure With Signaling Domain

FoxA transports ferrioxamine B (Feox) across the OM of *P. aeruginosa* and transmits a signal across the OM into the periplasm, which initiates the transcription of Feox transport genes (Table 1) (Schalk and Perraud 2023). FoxA has a typical TBDT structure with an N‐terminal extension involved in transcriptional regulation. Three crystal structures were determined: FoxA loaded with Feox, FoxA unloaded (apo Fox), and FoxA in complex with the C‐terminal domain of TonB (TonB_Ct_ residues 251–340) (Josts, Veith, and Tidow 2019). In the loaded structure, the large lumen faced the extracellular side of the membrane. No electron density was observed in the N‐terminal signaling domain (residues 45–143), most likely because of the high flexibility of the linker connecting the plug and signaling domains, as observed previously in FecA (Ferguson et al. 2002; Yue, Grizot, and Buchanan 2003) and FpvA (Brillet et al. 2007; Greenwald et al. 2009; Wirth et al. 2007). In apo FoxA, most of the TonB box was occluded in the interior of the barrel. In the FoxA TonB_Ct_ complex the TonB box was displaced approximately 22 Å from the folded plug domain to bind TonB. In FoxA with bound Feox and TonB_Ct_, loops 7 and 8 were displaced by 7 Å on the extracellular side of the OM, which closed the barrel lumen on both sides of the OM and prevented translocation of Feox through the channel. Isothermal titration calorimetry of TonB_Ct_ in apo FoxA reconstituted into nanodisks, revealed strong saturable exothermic heat, indicating tight binding between TonB_Ct_ and apo FoxA. No such binding was observed in similar experiments with TonB_Ct_ and FhuA (Mills et al. 2014) which is devoid of the signaling domain. Feox increased 17‐fold the binding affinity between FoxA and TonB_Ct_. The results obtained with truncated versions of FoxA indicated that the stretch of amino acids located upstream of the TonB box mediates the constitutive mode of TonB_ct_ binding. Feox induces the release of the TonB box from within the barrel, resulting in a tight complex between FoxA and TonB.

#### 4.5.4 TBDT Substrates Other Than Fe Complexes and Vitamin B_12_

At the beginning of the TonB dependent transport studies Fe^3+^siderophores and vitamin B_12_ were used as transport substrates. But soon later other substrates were identified which will be discussed if new mechanisms were uncovered (Table 1).

#### 4.5.5 TonB‐Dependent Glycan Transport by *Caulobacter crescentus*

The genome of *C. crescentus* was predicted to encode 67 TBDTs, which were expected to fulfill functions other than the hitherto known transport of Fe siderophores. Since *C. crescentus* thrives in nutrient‐poor inshore waters, the nutritional value of the highly abundant oligosaccharides chitin and starch in nature was examined. *C. crescentus* grew on the chitin hydrolysis products N‐acetyl‐β‐d‐glucosamine (GlcNAc) up to the (GlcNAc)_5_ pentamer (Eisenbeis et al. 2008) and on the starch degradation products maltose up to maltohexaose (Neugebauer et al. 2005). Transport of N‐acetyl‐β‐d‐glucosamines required the NagA OM protein and transport of maltodextrins required the MalA OM protein. Similar to Fe transport, transport of both sugars depends on TonB, ExbB, and ExbD; however, in contrast to Fe transport, GlcNAc and maltodextrin transport across the CM are not mediated by ABC transporters (Lohmiller et al. 2008). NagA (93 kDa) and MalA (96 kDa) were larger than *E. coli* TBDTs (average size 82 kDa) owing to the larger size of the surface loops. The genes encoding both OM proteins were flanked by genes encoding predicted transport functions across the CM. No change!

### 4.6 TBDTs With Accessory Proteins

#### 4.6.1 TonB‐Dependent Glycan Transport by Bacteroides

**FIGURE 6:**
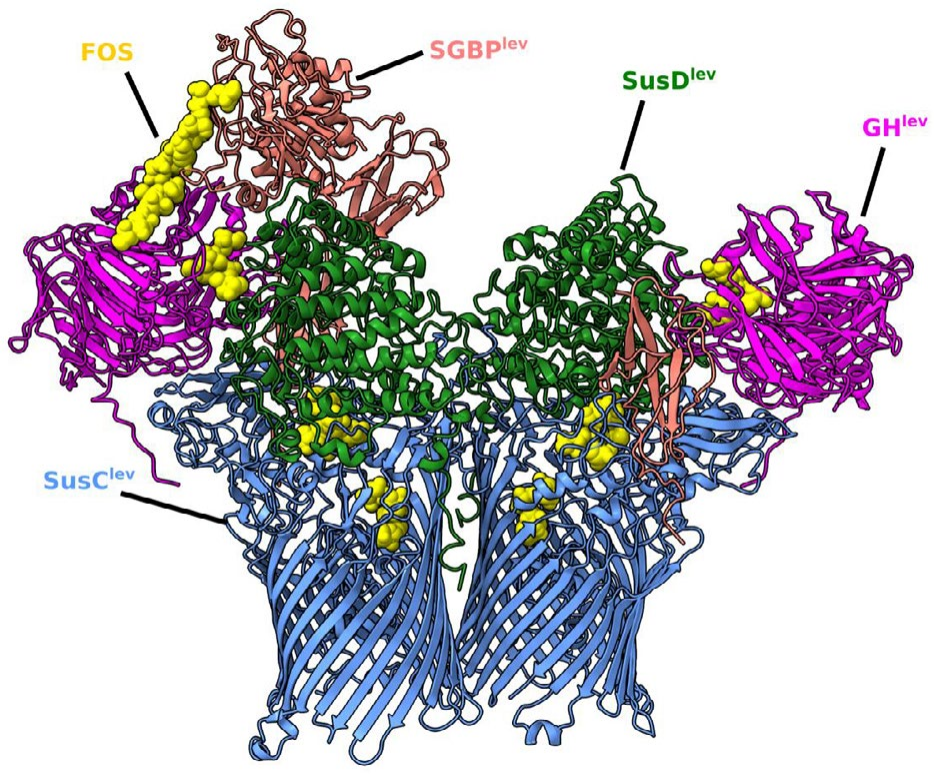
Utilisome of *Bacteroides thetaiotaomicron* for the use of levan as carbo source. The four dimeric proteins, Susc, SusD, GH and SGBP form a stable octamer in the outer membrane. SusC is the TonB‐dependent transporter (TBDT) to which SusD is associated. SusD caps the extracellular face of SusC and alternatively exposes and occludes the glycan‐binding sites within the SusC barrel. The levan degradation product (colored yellow) consisting of six β2,6‐linked fructose units (FOS) with a β2.1 decoration on fructose‐4 is located at the interface between FusC and FusD and shown as it moves across the complex (PDB 8AA2) Figure was provided by Bert van den Berg (Silale and van den Berg 2023).

Bacteroides is the dominant Gram‐negative genus in the human gastrointestinal tract. They thrive on a wide variety of polysaccharides that human hosts cannot digest. One species may encode more than 100 polysaccharide utilization loci (PULs), of which each encodes a complete set of proteins to degrade polysaccharides into smaller oligosaccharides and transport them into the cells (Salyers et al. 1977; Reeves et al. 1996; Glenwright et al. 2017; Pollet et al. 2021; Silale and van den Berg 2023; Parker et al. 2022). Surface‐exposed lipoproteins (SLP) include ligand‐binding proteins (SusD), glycoside hydrolases (GH), surface‐binding proteins (SGBPs), and proteases. The dimeric SusCD pair represents a transporter to which one GH and one SGBP are attached (Figure 6). SusD contains two oligosaccharide‐binding sites and assumes three distinct states, depending on the positioning of its lid: open–open, open–closed, and closed–closed. The oligosaccharides are initially bound to SGBP and then transferred to GH; after fragmentation into smaller units, they are transported by SusC across the OM. SusC has the typical TBDT structure with a TonB box and requires TonB, ExbB, and ExbD to translocate sugar substrates from the cell surface into the periplasm. Bacteroides encode many more TBDTs than the energy‐transducing proteins TonB and ExbB‐ExbD. Notably, of the six predicted TonB proteins in *Bacteroides fragilis* only TonB3 is involved in the import of starch, N‐linked glycans, mucin‐glycan degradation products, heme, ferrichrome, and vitamin B_12_ (Parker et al. 2022). As the amino acid sequence of TonB boxes is flexible, a single TonB protein functionally interacts with many TBDTs as long as it assumes a certain secondary structure.

### 4.7 TonB‐Dependent Transport of Proteins Through TBDTs

#### 4.7.1 TonB‐Dependent Uptake of Colicin M by the TBDT FhuA: Double Interaction of TonB With the TBDT and Colicin

B‐group colicins such as colicins B, D, M, Ia, and Ib are taken up by cells via a TonB‐dependent mechanism (Cascales et al. 2007). The uptake and mode of action of Cma have been studied in detail (Braun, Helbig, and Patzer 2012) and are discussed here as an example of TBDT‐mediated protein import. Colicin M (Cma) is encoded on the plasmids of certain strains of *E. coli* and, more rarely, on the chromosomes of clinical isolates. Once secreted by the producing cells, Cma kills *E. coli* cells that do not produce cognate immunity protein. The lysis of sensitive cells results from the inhibition of peptidoglycan biosynthesis (Schaller, Holtje, and Braun 1982; Harkness and Braun 1989) through the release of C_55_‐OH (undecaprenol) from the lipid II peptidoglycan precursor C_55_‐PP‐MurNac(−pentapeptide)‐GlcNAc (El Ghachi et al. 2006). Cma phosphatase activity is unique among the colicin toxins. Cma is composed of an N‐terminal translocation domain, a central receptor‐binding domain, and a C‐terminal phosphatase domain (Dreher, Braun, and Wittmann‐Liebold 1985; Zeth et al. 2008). The intrinsically unstructured N‐terminal translocation domain contains the TonB box ETLTV at positions 2–6 which is required for Cma uptake across the OM into the periplasm (Köck et al. 1987; Pilsl et al. 1993). Mutations L4P, L4N, V6E, V6K, and V6R in the TonB box inactivate Cma. Their activity is restored by the mutation Q160L in TonB. Inactive FhuA TonB box mutants become active by the same TonB mutant (Schöffler and Braun 1989). Cma uptake requires interaction of TonB with the TonB box of Cma and FhuA.

FhuA in nanodisks forms a high‐affinity binding site for Cma (approximately *K*_*D*_ 3.5 nM). When incubated with CmA and TonB, FhuA forms a single band after native polyacrylamide gel electrophoresis, which dissociates into FhuA‐Cma and free TonB upon gel filtration. Moreover, the addition of TonB to FhuA‐Cma did not change heat production, indicating that Cma did not promote the interaction between FhuA and TonB (Mills et al. 2014).

The following scenario is envisioned for the uptake of Cma by FhuA: binding of Cma induces structural transitions in FhuA such that the TonB box of FhuA comes into contact with TonB. After activation by a pmf through ExbB‐ExbD and TonB, the FhuA plug moves and opens the pore, enabling Cma to move to a site where its TonB box binds to TonB. This interaction translocates Cma to the periplasm. To fit into the open pore, Cma unfolds and refolds in the periplasm by FkpA, a peptidyl prolyl *cis‐trans* isomerase/chaperone (Hullmann et al. 2008; Helbig et al. 2011). FkpA is essential for Cma activity soon after Cma enters the periplasm. It can be envisioned that in a FhuA TonB box mutant or a Cma TonB box mutant, CmaA would be trapped in the pore and provide insights into the transfer steps, including the structural changes in FhuA, Cma, and TonB, and the sequence of interactions between the three proteins.

### 4.8 TonB‐Dependent Transport of Cma Homologs

The activity domain of Cma is found in several bacteriocins from different species (Chérier et al. 2021) such as *E. coli*, *Shigella*, *Yersinia* and the family of Pasteurellaceae, but the Cma homologs differ in the receptor‐binding and translocation domains. Bacteriocin is the general name for colicin‐like protein toxins and includes those produced by strains that do not belong to *E. coli*. For example, *Pectobacterium* spp. produce pectocin M1 and M2, which contain a ferredoxin‐binding domain that is connected by a flexible α‐helix to the Cma‐like cytotoxic activity domain. The ferredoxin domain is homologous to plant ferredoxins and is used by *Pectobacterium* to scavenge Fe during plant infections (Grinter, Milner, and Walker 2012). FusA serves as a receptor through which pectocin enters *Pectobacterium atrosepticum* (Grinter et al. 2016). FusA is a member of the TBDT protein family with highly extended loops that form a large binding surface for the ferredoxin molecule. The periplasmic FusC protease probably cleaves the ferredoxin moiety, and the released Fe‐sulfur cluster is imported across the CM by the FusDBC transporter and used as a Fe source.

### 4.9 TonB‐Dependent Transport of the Bacteriocin Pyocin Through FpvAI

TBDT FpvAI transports ferric pyoverdine (FePvd) across the OM of *P. aeruginosa* in a TonB‐dependent manner (Table 1). It also serves as a receptor for pyocin S2, a 74‐kDa endonuclease that enters and kills *P. aeruginosa* cells (McCaughey et al. 2016). The 23 kDa N‐terminal fragment of pyocin (pyoS2^NTD^) outcompetes FePvd bound with high affinity to FpvAI (*K*_*d*_ = 240 pM) (White et al. 2017). In the crystal structure, the proline‐rich region (PRR, residues 33–45) of pyoS2^NTD^ primarily contacted the FpvAI plug domain, occupied the same binding site within the plug, and assumed a shape similar to that of FePvd. Similarly, pyoS2^NTD^ induced conformational changes within the plug, similar to those triggered by FePvd. FpvAI served as a receptor, as the cells incubated with pyoS2^NTD^ labeled with Alexa Fluor became fluorescent. Fluorescence recovery after photobleaching indicated rapid diffusion into the periplasm because dissipation of the pmf by the protonophore CCCP prior to labeling with pyoS2^NTD^–Alexa Fluor 488 resulted in no fluorescence recovery after photobleaching. CCCP inhibited the uptake of pyoS2^NTD^–Alexa Fluor 488 into the periplasm, and the labeled protein remained bound to FpvAI in the OM of deenergized cells. Of the three *tonB* genes in *P. aeruginosa* only TonB1 functions in pyoS2 uptake by interacting with the TonB box MVITH (residues 11–15) of pyoS2. In the absence of the N‐terminal 21 residues, pyoS2^NTD^–Alexa Fluor 488 remained bound to the cell surface, where it could be degraded by trypsin.

The route taken by pyoS2 through FpvA1 was deduced from the crystal structure of the FpvAI‐pyoS2^NTD^ complex (White et al. 2017). GFP was attached to the C‐terminus of pyoS2^NTD^ which prevented the uptake of the fusion protein into the periplasm, thus “immobilizing” pyoS2^NTD^ close to the cell surface. Crosslinking experiments revealed that pyoS2^NTD^ moved quite a distance into FpvA1 because Gln184 of pyoS2^NTD^ was located close to the entrance of the plug crosslinked to Val197 of FpvAI at the center of the plug. Four different residues of pyoS2^NTD^ were cross‐linked to the same FpvA plug domain residue, Met177, suggesting that much of pyoS2^NTD^ passed through this residue.

The plug domain of FpvA1 is composed of force‐labile and force‐resistant subdomains, as has been demonstrated for FhuA and BtuB (Hickman et al. 2017). The following translocation steps of pyoS2 across FpvA1 can be envisioned: pyoS2 binds to FpvA1, which induces structural changes in FpvA1 and pyoS2, including the recruitment of the C‐terminal domain of TonB1 in the periplasm. The interaction of the FpvA1 TonB box with TonB1 creates a mechanical force derived from the pmf through the TonB1‐ExbB_5_‐ExbD_2_ complex, which unfolds the force‐labile FpvA1 plug subdomain. This creates an approximately 13 Å‐wide channel through which the N‐terminus of pyoS2, including the TonB1 box, moves into the periplasm, where it engages with TonB1. The pyoS2 is then translocated through the FpvA1 channel into the periplasm in a pmf‐dependent manner.

### 4.10 TonB‐Dependent Uptake of Bacteriocins via Receptors That Do Not Belong to TBDTs

*Klebsiella* strains produce klebicin (KlebC), which enters cells via unusual mechanisms. TolC functions as a protein import channel for KlebC, although it is part of the efflux pumps through which *E. coli* removes toxic compounds (Koronakis 2003). KlebC contains an N‐terminal elongated helical hairpin that binds to TolC (Housden et al. 2021). The helical hairpin opens like a switch blade to bind TolC. The cryo‐EM structure of the KlebC derivative reveals a partially translocated molecule associated with the length of the TolC channel. The unstructured N‐terminus of KlebC protrudes into the periplasm, where it binds through its TonB box to TonB, which is coupled by ExbB‐ExbD to the pmf. The pmf drives the import of klebicin through this channel.

The KlebC uptake model probably applies to the uptake of colicins 5 and 10. They bind to Tsx, a nucleoside‐specific porin (Hantke 1976) that serves as a receptor for phage T6 and colicin K. TolC is essential for the uptake of colicins 5 and 10. Furthermore, colicins 5 and 10 contain a TonB box and require TonB to kill cells (Pilsl and Braun 1995b, 1995a). Tsx and TolC do not contain TonB boxes and they do not belong to the TBDT class. Both colicins share 55% sequence identity with KlebC in the N‐terminal domain which is an important region for TolC recognition. The crystal structure of Tsx consists of a monomeric 12‐stranded β barrel with a long and narrow channel spanning the OM (Ye and van den Berg 2004). The single‐channel conductance of Tsx at 10 pS is much smaller than that of the LamB channel (160 pS) and the general *E. coli* diffusion pore OmpC (1900 pS) (Benz et al. 1988). Colicins 5 and 10 do not fit into a pore with a diameter of Tsx; it is too small for the transfer of proteins of sizes of colicins 5 and 10. Tsx likely serves as the initial binding site from which colicins are translocated to TolC, through which they are transferred across the OM into the periplasm.

### 4.11 TonB‐Dependent Uptake of Pyrroloquinoline‐Quinone by *E. coli*

*E. coli* induces under phosphate‐limiting conditions the pyrrolochinoline‐quinone (PQQ)‐dependent glucose dehydrogenase and glucose may be oxidized to gluconolactone in the periplasm. The hydrolysis product gluconate appears in the medium and solubilizes phosphate salts and is used as carbon source. As *E. coli* does not produce PQQ de novo, it requires external PQQ, which is transported from the medium into the periplasm by TBDT PqqU (previously designated as YncD) in a TonB‐dependent manner (Table 1) (Hantke and Friz 2022). PqqU has a high affinity for PQQ and 1 nmol PQQ is generally sufficient for growth. This transporter is widespread in environmental bacteria, with orthologs sharing between 33% and 99% identity (McIntire 1998; Southall et al. 2006). The most highly conserved residues are between amino acids 79 and 150. The crystal structure revealed a typical TBDT (Grinter and Lithgow 2020).

### 4.12 TonB Dependent Secretion of the PopC Protease by the TBDT Oar

*Myxococcus xanthus* initiates a multicellular developmental program in response to starvation, leading to the formation of fruiting bodies inside which rod‐shaped cells differentiate into spores. Starved cells secrete PopC, which accumulates in the periplasm prior to secretion. Secretion depends on the Oar protein which exhibits a typical TBDT structure, with an N‐terminal TonB box and a 22‐stranded β barrel with a diameter of 35–40 Å enclosing a plug (Gómez‐Santos et al. 2019). Notably, *exbB1* and *exbD1* are adjacent to the *oar* gene, and secretion requires pmf, as experimental dissipation of pmf reduced the amount of PopC in the supernatant. Mutants of *tonB1* blocked PopC secretion, whereas mutants of *exbB1, exbD1, exbD2* displayed aberrant development but still secreted reduced amounts of PopC. As one of the additional three *tonB* genes and six *exbB exbD* genes in *M. xanthus* may have complemented the function of TonB1‐ExbB‐ExbD1‐ExbD2, Oar‐dependent PopC secretion was studied in wild‐type *E. coli* expressin*g* single TonB, ExbB, and ExbD proteins. When co‐expressed with Oar, PopC was exclusively detected in the supernatant of *E. coli* cell culture. The *E. coli* Ton system energizes Oar to secrete PopC. PopC secretion also demonstrated that a substrate as large as 50.8 kDa could pass through the pores of TBDT. It would be interesting to unravel the mechanistic differences between secretions and imports.

### 4.13 Movement of the Plug Domain by Mechanical Force

To transfer substrates through TBDTs, the plug domain must be fully or partially unfolded to open the lumen. In the pulling hypothesis, TonB binds to the TonB box of TBDTs and applies a mechanical remodeling force driven by pmf. The interaction between the TonB box and TonB must be sufficiently strong under tension to allow the unfolding of the plug domain before TonB dissociates from the TBDTs. The complex between the soluble TonB (33–239) fragment, in which the N‐terminal transmembrane helix was deleted, and the TonB box of BtuB was studied using single‐molecule force spectroscopy (Hickman et al. 2017). The data indicated that the inter‐polypeptide interactions between TonB (33–239) and BtuB were stronger than the intra‐polypeptide interactions in the plug domain. The plug unfolded before the TonB (33–239) BtuB complex was dissociated. The TBDT‐TonB complex was sufficiently durable under force to allow the unfolding of half of the plug domain before its dissociation. Fifty residues (numbers 23–73) downstream of the TonB box most likely unfold first. This mechanically weak plug domain creates a channel between the plug and the barrel. Complete unfolding allows entry of large antibiotics, such as bacitracin. Based on these and additional data it is proposed (Hickman et al. 2017) that the plug domain is divided into two separate functional parts: a mechanically strong subdomain involved in signaling and a mechanically weak subdomain that is unfolded to allow substrate transport. The mechanically strong subdomain may prevent increase of OM permeability for toxic compounds.

#### 4.13.1 Transfer of Energy From the pmf Across the Periplasm to TonB

The pmf provides energy for the active transport of TBDTs across the OM (Hancock and Braun 1976). The response of TonB to pmf was completely obscure for a long time. A genetic locus termed *exbB* participated in TonB‐dependent functions (Guterman and Luria 1969; Guterman 1973). However, *exbB* mutants exhibited residual activity and were therefore considered to encode auxiliary functions. In fact, *exbBD* mutants are fully sensitive to phages T1 and φ80 (Hantke and Zimmermann 1981). DNA sequencing of the *exbB* locus revealed two adjacent genes, *exbB* and its downstream *exbD* (Eick‐Helmerich and Braun 1989). The 26 kDa ExbB protein starts with the N‐terminus in the periplasm and then crosses the CM thrice. The 17.8 kDa ExbD protein is anchored by the N‐proximal region in the CM and extends into the periplasm (Kampfenkel and Braun 1992, 1993; Ahmer et al. 1995). The 26% sequence identity between ExbB and TolQ and the 25% sequence identity between ExbD and TolR suggested similar functions and prompted comparison of the properties of mutants. The *exbB* mutants that are partially resistant to albomycin and colicins B, D, and M are fully resistant after introduction of an additional *tolQ* mutation (Braun 1989; Braun and Herrmann 1993). Wild‐type TolQ complemented the mutant ExbB. These findings are supported by studies of the transport of vitamin B_12_ into the periplasm (Bradbeer 1993). The transport rate into the *exbB* mutant was 20% of that of the *exbB* wild‐type strain and below 5% in the *exbB tolQ* double mutant. Complementation of the double mutant by *tolQ* restored the transport. In addition, complementation with wild‐type *exbB* resensitized *tolQ* mutants to colicins E1 and E2 which require active TolQ to enter the cells (Samire et al. 2020). Cross complementation between ExbB‐ExbD and TolQ‐TolR explained the residual activities of the *exbB exbD* mutants in TonB dependent functions and moved the “auxiliary” ExbBD functions into the center of the pmf‐driven transport by TBDTs across the OM.

### 4.14 Interaction of TonB, ExbB and ExbD

The physical interaction between TonB, ExbB, and ExbD was observed by co‐elution of ExbD and TonB together with ExbB fixed as a (His)_6_ derivative on a Ni‐NTA column. Spontaneous degradation of TonB was prevented by co‐expression with ExbB, which also prevented the degradation of ExbD. The degradation of TonB by trypsin and proteinase K was not inhibited by ExbB, which however prevented trypsin degradation of ExbD (Fischer, Gunter, and Braun 1989; Ahmer et al. 1995). These data indicate that ExbB binds to TonB and ExbD. Solubilization of ExbB and ExbD by the detergent undecyl maltoside yielded a mixture of ExbB homo‐oligomers and ExbB‐ExbD heterooligomers, of which the main components ExbB_6_‐ExbD1 and ExbB_5_‐ExbD1 formed stable oligomers upon further purification (Pramanik et al. 2010, 2011). No TonB was present in these preparations, and the subsequent ExbB‐ExbD isolates used for cryo‐EM. However, in the 3.8 Å resolution structure of the ExbB‐ExbD complex of *Pseudomonas savastanoi* a rod‐like structure was observed that corresponds to a single transmembrane helix of the TonB subunit (Deme et al. 2020).

Single‐particle cryo‐electron microscopy has revealed the structure of the ExbB‐ExbD complex (Celia et al. 2019; Biou et al. 2022, 2023; Ratliff, Buchanan, and Celia 2022; Maki‐Yonekura et al. 2018). Protein complexes were isolated from *Serratia marcescens* and *E. coli*. The following discussion is mainly based on *S. marcescens* data, which have recently provided the most advanced insights (Biou et al. 2022, 2023; Zinke et al. 2024). However, *E. coli* proteins have been studied in parallel and have yielded similar results. *S. marcescens* transports heme across the OM through HasR, a TBDT (Wandersman and Delepelaire 2012; Cescau et al. 2007). The heme is tightly bound to a small protein designated as a hemophore (HasA), which binds to HasR. Upon binding, the heme dissociates from the hemophore and translocates across the OM into the periplasm. Transport across the OM is energized by the TonB paralog, HasB, which is specific for heme uptake, and by ExbB‐ExbD. Compared to *E. coli* ExbB, *S. marcescens* ExbB contained an N‐terminal extension of 81 residues that did not appear in the cryo‐EM structure. Deletion of the N‐terminal extension yields an *S. marcescens* ExbB derivative that functions with HasB and TonB in *E. coli*. However, the growth of cells harboring ExbB with an N‐terminal deletion was delayed for 2 h compared with that of cells carrying wild‐type ExbB. Although not essential, the N‐terminal extension contributes to ExbB activity and is frequently found in ExbB orthologs, which are most similar in the region forming the transmembrane channel, whereas the residues located at the membrane surface are more variable.

In the isolated ExbB‐ExbD complex, ExbB forms a pentamer enclosing a pair of ExbD transmembrane segments (Figure 1) (Biou et al. 2022). Helices 2 and 3 of each ExbB monomer create a large hydrophobic cavity comprising a central nonpolar pore. At the center of the transmembrane segment, ExbD contains an aspartate residue (Asp25), which is predicted to play an essential role in proton transfer, similar to the Asp residue in P‐type ATPases (Eick‐Helmerich and Braun 1989). In the cryo‐EM model, Asp25 of chain G faces Thr218 of chain C, while Asp 25 of chain F faces the interface between the two ExbB monomers. The estimated pKa values of 7.3 and 7.4 allow protonation and deprotonation at physiological pH. Compared to the ExbB structure, ExbB‐ExbD shows a 4.3 Å opening toward the periplasmic side. In ExbB‐ExbD of *S. marcescens* two channels extending from the periplasmic entrance to Asp25 cross the membrane with an average diameter of 3 Å, thereby connecting the cytoplasm to the periplasm and potentially serving as the proton trajectory.

The central transmembrane pore domain is not located in the CM but shifted about 10 Å toward the periplasmic space which positions the dimer of the transmembrane helix of ExbD above the membrane plane (Figure 1). Consequently, the central cavity of the ExbB pentamer expands almost halfway into the membrane plane, and the Asp25 residue is in close proximity to the CM periplasmic leaflet (Ratliff, Buchanan, and Celia 2021; Buchanan et al. 1999).

### 4.15 The Important Role of the Periplasmic Domain of ExbD

Cryo‐EM cannot resolve the structure of the ExbD periplasmic domain because of its dynamic and disordered nature. Therefore, NMR was used to determine the structure of the soluble periplasmic domain of the *S. marcescens* ExbD (residues 43–140), which revealed that this domain forms a dimer with each monomer consisting of a four‐stranded β‐sheet and two α‐helices (Zinke et al. 2024). Part of the dimeric interface is formed by a swapped intermolecular and antiparallel β‐sheet, which has been designated the N‐terminal Intermolecular β‐Strand (NIBS, residues 44–49). NIBS connects intramolecular β‐sheets across the dimer interface. The equivalent ExbD fragment from *E. coli* was only soluble at pH 3, monomeric, and displayed disordered NIBS (García‐Herrero and Vogel 2005). ^15^N‐chemical exchange saturation transfer profiles revealed that the NIBS of ^15^N‐labeled ExbD transitioned through three different states: a folded closed state, a disordered open state, and an intermediate state (Zinke et al. 2024). Residue V47 is located at the dimer interface in the closed state. A cysteine residue locked the periplasmic fragment in the closed state by inducing the formation of a disulfide bridge. Cells expressing ExbD V47C displayed reduced heme‐dependent growth rates. Reduced growth was also observed in the ExbD V47G and ExbD V47R mutants, in which ExbD mainly assumed an open state. The main conformation of ExbD is a closed state, in which the proton channel is closed. The open form is sparsely represented and selected upon the binding of TonB to an intrinsically disordered region, which then undergoes a disorder‐to‐order transition. Open state ExbD preferentially binds to TonB. The TROSY spectra of ^15^N‐labeled HasB and ^15^N‐labeled TonB changed upon the addition of unlabeled ExbD. ExbD interacts with the intrinsically disordered NIBS region of HasB close to the N‐terminal α‐helix. The site of interaction was confirmed by the crystal structure of ExbD lacking residues 43–60 in complex with TonB peptide (residues 35–56), which was localized between the two ExbD protomers, thereby replacing the lacking NIBS residues.

Previous cross‐linking experiments indicated the importance of the periplasmic domain of ExbD. Formaldehyde dimerizes and connects ExbD to TonB and ExbB (Ollis et al. 2009). The periplasmic regions of ExbD (residues 92–121) and pmf are important for building homodimers and heterodimers and for crosslinking the periplasmic domains of TonB with ExbD, respectively. The failure of the inactive mutants ExbD(D25N) and TonB(H20A) to crosslink indicates the relevance of cross‐linking data for understanding the functional interactions of the three proteins. When replaced by cysteines, ExbD residues A92, K97, and T109 are cross‐linked to multiple substituted residues in the TonB carboxy‐terminus. Single amino acid substitutions of the three cytoplasmic residues decreased ExbB activity, but still crosslinked ExbB into tetramers (Jana, Manning, and Postle 2011). They failed to support pmf‐dependent formaldehyde crosslinking between the periplasmic domains of TonB and ExbD, suggesting signal transduction from the cytoplasm to the periplasm. Further cross‐linking studies revealed that the region between residues 42 and 63 is highly disordered, flexible, and essential for signaling to the periplasmic C‐termini of ExbD and TonB. Single alanine replacement of residues V45, V47, V49, and P50 decreased but did not inactivate TonB‐related activities, such as Fe‐ferrichrome and Fe‐enterobactin transport (Kopp and Postle 2020). However, double‐mutant combinations were inactive. These results and many other cross‐linking data indicate that ExbD couples TonB to the pmf, with the concomitant transition of ExbD and TonB periplasmic domains from unenergized to energized heterodimers. In this context, ExbB serves as a scaffolding protein for the assembly of ExbD and TonB (Ollis et al. 2009; Ahmer et al. 1995).

### 4.16 ExbD Is Fixed to the Peptidoglycan

The location of the ExbD C‐terminal domain in the periplasm prompted the investigation of the interaction between ExbD and peptidoglycan by NMR (Zinke et al. 2024). The [^1^H‐^15^N]‐TROSY spectra of the ^15^N labeled periplasmic domain of ExbD indicated the disappearance (broadening) of peaks upon the addition of peptidoglycans isolated from *E. coli* and *Staphylococcus aureus*. In contrast, the peptidoglycan of *Bacillus subtilis* did not change its spectra, indicating that ExbD does not bind to peptidoglycans with nonacetylated glucosamine moieties. Differences in the peptidoglycan side chains of *E. coli* and *S. aureus* did not affect ExbD binding. The decreased peak intensities were mapped to the ExbD dimer interface, which was significantly reorganized upon binding to the peptidoglycan. ExbD, with cross‐linked V47C residues, failed to dissociate the dimers and showed no peptidoglycan‐induced spectral changes. Transient fixation of the ExbD dimer to a peptidoglycan may serve as an anchor for the pmf‐dependent movement of ExbB and TonB.

### 4.17 Tentative Sequence of Events During TBDT Mediated Transport

The following discussion is mainly based on recent results obtained with the heme transport system of *S. marcescens* (Biou et al. 2023; Zinke et al. 2024) but may be applicable to all TBDT‐mediated OM transport systems. ExbD in the ExbB‐ExbD complex primarily assumes a closed conformation with proton‐impermeable pores. The closed form is in equilibrium with the rare open form. Interaction with peptidoglycan results in the loosening of the dimer interface. TonB enters between the two ExbD monomers and its intrinsically disordered region (IDR, residues 46–61) binds to ExbD. In the resting state, TBDT is not loaded with a ligand. Ligand binding triggers the movement of the TBDT N‐terminal domain into the periplasm, where TonB binds to the TonB box of TBDT. The pmf‐induced rotation of ExbB induces the rotation of TonB bound to ExbB, resulting in a pulling force on the plug that opens the TBDT and enables active transport of the ligand. It is not known how pmf induces the rotation of ExbB via protonation and deprotonation of Asp25 in ExbD. Ratliff, Buchanan, and Celia (2022) proposed that the disordered linker of TonB wraps around ExbD and exerts a pulling force on the TonB box of TBDT, resulting in the movement of the plug toward the periplasm (Ratliff, Buchanan, and Celia 2022). If force generation by MotA‐MotB, the flagellar motor that propels swimming bacteria (Wadhwa and Berg 2022) is used as a model, ExbB rotates because the homologous MotA rotates. However, if the proton flux drives rotation, ExbD rotates. The movement of the plug releases the ligands from their binding site on TBDT into the periplasm, and the system returns to the resting state, as previously illustrated (Braun et al. 2023).

Although force generation by rotation is at present the favored mechanism (Rieu et al. 2022; Webby et al. 2022; Williams‐Jones et al. 2023) other mechanisms are considered. In the TBDT crystal structures ligands are bound to plugs. Ligand transfer into the periplasm may involve pulling of the plugs into the periplasm (Hickman et al. 2017). In the wrap and pull mechanism rotation of the ExbD dimer leads to the wrapping of the TonB linker around ExbD leading to the pulling of the TonB C‐terminal domain to the TBDT TonB box (Ratliff, Buchanan, and Celia 2021, 2022).

### 4.18 The Three Motors ExbB‐ExbD, TolQ‐TolR, and MotA‐MotB Generate Energy, and TonB TolA Transmit the Energy From the Cytoplasmic Membrane to the OM by Similar Mechanisms

All three devices, ExbB‐ExbD, TolQ‐TolR, and Mot‐MotB, form pentamers encircling pores that contain protein dimers. They function via conserved force generation and transduction mechanisms. The complex reaction sequence of TolQ, TolR, TolA, TolB, and Pal maintains the OM integrity during cell division (Webby et al. 2022). The TolQ‐TolR‐TolA complex accumulates at the site of cell division, where TolB‐Pal diffuses through the periplasm along the inner side of the OM. In the force‐requiring reaction, TolA releases Pal from TolB. Pal accumulates at the division site where it connects the three layers of the cell envelope through a pmf‐dependent process, thereby maintaining OM integrity. The bacterial flagellar motor drives the flagella and generates propulsion through the rotation of helical extracellular filaments. The rotation is driven by the H^+^ influx across the CM, which is driven by pmf. The three motor subunits are related to each other by a 10°–16° rotation, which supports rotary motion as a means of force generation (Williams‐Jones et al. 2023). The TonB and TolA force transducers associate with the ExbBD and TolQR motors, respectively, and span the periplasm to contact the TBDTs or TolB, thereby converting the pmf into mechanical work at the OM. Their central domains form elongated hairpin structures and do not share a sequence similarity. TonB forms a type II polyproline helix (Domingo Köhler et al. 2010) while TolA forms an extended α helix (Levengood, Beyer Jr., and Webster 1991). As bridging the periplasm to transmit force from the CM to the OM is their main function, the central domains were exchanged between TonB and TolA, with the expectation that they would complement each other. Both chimeras were indeed active; TonB carrying the TolA segment rendered the cells sensitive to colicin Ia, which requires TonB to enter the cells. Cells expressing TolA with the TonB segments are sensitive to Tol‐dependent colicin E9 (Williams‐Jones et al. 2023). The TolA hybrid also stabilized the OM, which resisted exposure to 0.2% SDS, whereas TolA wild‐type cells grew in the presence of 2% SDS. TonB and TolA construct chimeras with different insert sizes restored the functions of the *tonB* and *tolA* mutants to various degrees, indicating that different levels of force were created, which affected colicin uptake and OM stability to various degrees. Complementation of *exbB exbD* mutants by *tolQ tolR* genes and vice versa already had demonstrated that the complex TolQ‐TolR is able to activate TonB and ExbB‐ExbD, which is able to activate TolA (Braun and Herrmann 1993).

## 5 Perspective

The existence of energy‐consuming transport systems in a membrane with no energy source was an exceptional insight gained by studying TonB‐mediated activation of TBDTs. Confronted with this extraordinary fact, one could expect novel mechanisms for substrate translocation across the OM. Indeed, new methods of protein activity regulation have been uncovered. The TBDTs form β barrels with plugs that close the central lumen of the β barrels. Pmf of the CM, mediated by the TonB‐ExbB‐ExbD protein complex, opens the plug of the TBDTs. Highly specific and tight substrate binding to the TBDTs at the cell surface induces local allosteric transitions that propagate across the entire plug up to the periplasm. All TBDTs contain a conserved homologous N‐terminal pentapeptide, designated TonB box, which is the principal site of interaction with TonB. Various spectroscopic methods and chemical cross‐linking revealed multiple structural changes and interactions between the plug and the TonB‐ExbB‐ExbD proteins which depended on the presence of substrate and pmf. Crystal structures of various TBDTs and cryo‐EM structures of ExbB_5_‐ExbD_2_ oligomers formed the basis for the device and interpretation of biochemical and spectroscopic experiments. With different samples, purified proteins, proteins in membrane fragments, proteins solubilized in detergents, isolated proteins reconstituted in lipid bilayers and nanodisks, major insights into the dynamics of the protein interactions were obtained. Current models propose that energy derived from pmf generates mechanical force to open the plug. It remains to be experimentally proven how the pmf serves as an energy source, how energy is transferred to the ExbB_5_‐ExbD_2_ complex and how interaction of “energized TonB” opens the TBDT pore. Since there are many more TBDT molecules than TonB molecules in an OM it is unknown how TonB selects substrate‐loaded TBDTs. If genomes encode several *tonB* genes frequently only one is used for the transport of all of the examined substrates. The function of the remaining TonBs is unknown. TBDTs transport a wide range of substrates, metal ions, metal complexes, sugars, vitamins, and proteins which are too large to diffuse through the porins or are only available in very small amounts. The import mechanisms may differ from the hitherto studied model compounds, Fe^3+^ siderophores, heme and vitamin B_12_. The TBDTs that transport oligosaccharides are associated with a second protein that delivers the substrate. Uptake of colicins require in addition to the TBDT TonB box an additional TonB box in the colicins. Some proteins are TonB‐dependent transported across the OM by proteins that do not belong to the TBDT class. In addition to the involvement of TonB in various types of import systems TonB is also required for the export of a protease.

Many TBDTs regulate the transcription of transport genes. Regulating TBDTs are equipped with a signaling domain at the N‐terminal end. Upon binding of a substrate, structural changes in the plug domain are initiated, which require TonB, TonB box, and the ExbB_5_ExbD_2_ complex. The signaling domain binds to the regulatory protein that extends from the periplasmic surface of the OM across the periplasm into the cytoplasm. The signal initiates proteolytic degradation of the regulatory protein resulting in a small N‐terminal fragment in the cytoplasm that activates the sigma factor which initiates transport gene transcription. Elucidation of the ways TonB‐TBDTs function in transcription regulation and import/export of substrates that differ from siderophores promises further insights into the sophistication of the TonB‐ExbB‐ExbD activities.

TonB is the essential component of systems by which bacteria respond to the composition of their environment. Binding of substrates to receptors opens pores through which the substrates pass through the otherwise impermeable OM. The ability to select substrates prevents entering of toxic compounds. Inspection of genomes predict many more TonB‐dependent transport and regulatory systems than are hitherto known.

## Author Contributions

**Volkmar Braun:** conceptualization, writing – original draft, writing – review and editing, formal analysis.

## Data Availability

Data sharing is not applicable to this article as no new data were created or analyzed in this study.
